# Health status and ergonomics education: A comparison between student nurses and first‐year nursing staff

**DOI:** 10.1002/nop2.2239

**Published:** 2024-07-11

**Authors:** Michal Hochhauser, Elena Liberman

**Affiliations:** ^1^ Department of Occupational Therapy Ariel University Ariel Israel; ^2^ Maccabi Health Services – Nursing Clinic Lev Ha'ir Medical Center Jerusalem Israel

**Keywords:** education, ergonomics, fatigue, musculoskeletal, training

## Abstract

**Aims:**

To (a) compare student nurses' health status and levels of ergonomics knowledge and awareness to those of first‐year nursing staff and (b) investigate the association between nurses' ergonomic compliance and health status with their educational preparedness.

**Design:**

This cohort study compared ergonomics awareness and knowledge, and health status of nurses when they were students and after their first‐year working in medical facilities.

**Methods:**

In total, 133 4th‐year student nurses completed a survey; 45 of them completed a second survey after working 1 year in a medical facility. Their health status was compared over time using repeated‐measures ANOVA. Correlation tests were used to analyse associations between ergonomics knowledge and awareness, health status, demographic variables and educational preparedness.

**Results:**

Respondents in both surveys displayed weak knowledge of ergonomic principles significantly associated with increased pain intensity and educational preparedness.

**Patient contribution:**

Ergonomics training should be expanded during nursing studies and first‐year training to prevent long‐term health disorders.

## 1 INTRODUCTION

Ergonomics is a science dealing with the functional interactions between people and their environment. Ergonomics principles and their impact on everyday activity, including work, receive insufficient exposure throughout the Western world, especially in medicine and nursing. Between 70% and 80% of European workers report discomfort due to nonneutral body postures (back or arm angles >30°) during repetitive work movements (Pheasant, 2003), and occupational diseases that tend to become chronic cause of 50% of work absences (Waters & Dick, 2015). This trend increased over the last 5 year. The percentage of nursing workers who incurred nonfatal occupational injuries increased from 7.1% in 2014 to 15.2% in 2019 (US Bureau of Labor Statistics, 2021). This scenario challenges health systems, requiring new strategies to manage workplace ergonomics and prevent occupational injury.

Musculoskeletal disorders (MSDs), pain, fatigue and other neurological signs such as migraine headaches are common problems that interfere with proper functioning in nursing. Throughout their studies, student nurses receive training in ergonomics to alleviate these difficulties. Nonetheless, clinical findings indicate a lack of knowledge and awareness of ergonomics and difficulty in applying this knowledge in clinical practice (Trinkoff et al., 2003).

### 1.1 Musculoskeletal disorders among nursing staff

Risks of MSDs affecting the musculoskeletal system's soft tissues and associated with nurses' work often occur due to ergonomic stressors. For instance, care in nursing homes includes dressing, bathing, feeding and toileting, actions involving close bodily assistance and multiple interactions that may injure the nursing staff (Trinkoff et al., 2003).

Psychosocial factors identified in nurses, such as job stress, inadequate staffing, job demands, insufficient support, time pressure and decision latitude, have been significantly associated with prevalence of musculoskeletal disorders in the wrists, shoulders, neck and hands (Zare et al., 2020).

Ergonomic damage can result from incongruity between work‐task demands and employees' ability to meet those requirements, especially in an overload situation. Muthukrishnan and Maqbool Ahmad (2021) found a significant association between nursing task‐specific risk‐exposure scores, ergonomic risk factors and the prevalence of MDs. The risk‐exposure scores were significantly associated with the reported MSDs of the lower‐back, neck and hip/thigh regions; these prevented routine activities at least once in the last 12 months. Ergonomic injuries can result during a single episode of overexertion, gradually over time from wear and tear, or a combination (Pheasant, 2003). Between 50% and 90% of intensive care nurses reported MSDs (Abedini et al., 2015; Ganiyu et al., 2015). Researchers found that standing for more than 3 h a day or walking/standing for more than 80% of a shift can cause lower‐back and leg pain (Waters & Dick, 2015). Monotonous work, heavy lifting, general patient‐care tasks (Lee et al., 2015) and poor body posture during demanding activities were found to cause long‐term MSDs and injuries (Kneafsey et al., 2012; Trinkoff et al., 2003) resulting in occupational disorders among nurses (e.g. intervertebral disc and sciatica damage, spondylitis, tendinitis, neuromuscular disorders and joint‐nerve diseases, such as tenosynovitis, carpal tunnel syndrome and tendonitis; Pheasant, 2003; Putz‐Anderson, 1988). Poor ergonomics leads to body aches, decreased satisfaction and work absences among nursing staff. These, in turn, can increase employees' physical and mental strain (e.g. working overtime) and the health system's loss of work capacity and unnecessary financial costs (Garzaro et al., 2022). Rectifying these problems requires increasing knowledge regarding proper ergonomics and promoting awareness of their importance in clinical practice during nurses' study and early career periods (Bernardes et al., 2022). Worldwide, researchers are investigating approaches to reduce the risk of disease and injury at work by integrating new assistive devices (Tamminen‐Peter & Fagerström, 2011) and developing and implementing new programs regarding ergonomics and safety (Kurowski et al., 2017; Pradap, 2015). Others are establishing physical‐training programs as effective interventions to improve neck movement and prevent or reduce muscle pain among nursing staff (Freimann et al., 2015).

Perplexingly, despite these new approaches and ‘zero‐lifting’ (nonmanual lifting) programs, occupational injury rates among nursing staff remain unchanged. When analysing ergonomic plans to minimize worksite risk factors, we can assume that increased MSDs may relate to a lack of personnel, poor training, increased work hours, pressure and stress. These factors allow nurses no time to think about proper body mechanics or rest during the workday.

Nurses have been taught according to guidelines to rest every 2 h at work, exercise and stretch before bed or chair transfers (The Israel Institute for Occupational Safety and Hygiene, and Ministry of Health, 2018). Nonetheless, medical institutions and nursing schools may provide insufficient guidance and practical training for all stages of the nurse's career, possibly due to a lack of administrative regulations of procedures and policies. In addition, nursing staff are unaware of potential long‐term health damage or solutions, such as personal ergonomic‐behavioural management (Lipscomb et al., 2004), including maintaining neutral posture during work and beneficially using body muscles and assistive equipment (Driessen et al., 2011; Nelson, 2006; Tamminen‐Peter & Fagerström, 2011).

In the United States, the National Nursing Examination (National Council of State Boards of Nursing; https://www.ncsbn.org) tests student nurses' basic knowledge of ergonomic principles for health practitioners. It assesses the basic application of body mechanics principles during patient care, medical devices and machinery use, and physical activity in nurses' personal and professional lives. However, scant research explored the connection between awareness and knowledge of ergonomics among (a) student nurses before starting their work and (b) new nursing staff. According to a current systematic review, evidence for the effectiveness of interventions in the nursing curricula for the prevention/treatment of physical complaints is scarce (Kox et al., 2020; Richardson et al., 2018). However, one recent study showed the effect of a comprehensive ergonomic program on student's knowledge, attitude and behaviour related to ergonomic standards in performing nursing interventions specifically ergonomic positioning (Prapti et al., 2020). Another study showed that the education of nursing staff about ergonomics can reduce the prevalence and risk of MSDs (Abdollahi et al., 2020). Therefore, this study sought to bridge that gap and compare health symptoms between these two periods and explore if educational preparedness facilitates awareness and reduces MSD's. The study assessed student nurses' awareness of ergonomic‐safety principles and medical ergonomics knowledge, including causes of musculoskeletal and occupational diseases, assistive devices and their operation prior to starting work in medical institutions. Additionally, this study examined whether the students' knowledge and awareness levels and health status changed after a 12‐month work period, as well as nurses' perception of *preparedness* for occupational challenges. Most of the research has investigated the cumulative aspect of symptoms in nurses who have been working long term; however, the current study wished to explore the affect of educational ergonomics preparedness of students and the impact after only first‐year of work. The findings may shed light on key ergonomic factors for education and training for novice nurses which can reduce occupational hazards and alleviate health risks at the start of their career, thus maintaining their jobs and job satisfaction.

### 1.2 Hypotheses

The research hypotheses were as follows:

Hypothesis 1 The first‐year nurses would demonstrate a significant decrease in their health status (e.g. body pain and orthopaedic diagnoses) compared to when they were student nurses.

Hypothesis 2 A significant positive association would be found between the presence of body pain and other health variables (e.g. migraines, BMI, stress and fatigue levels).

Hypothesis 3 No difference will be found in levels of knowledge of ergonomics principles and awareness between the student nurses and first‐year nurses. In first‐year nurses, differences in the number of medical diagnoses, the existence of migraine, fatigue, MSDs' and pain intensity would be found between those with lower levels of knowledge of ergonomics principles and awareness compared to higher levels.

Hypothesis 4 Educational curriculum will not suffice to enable student nurses and first‐year nurses to identify risk factors as perceived by them. A significant association will be found between this perception of first‐year nurses and adherence to ergonomic principles.

## 2 METHODS

### 2.1 Study design

In a prospective cohort study involving 133 student nurses, we administered a baseline survey to assess their knowledge and awareness of ergonomics principles. After 1 year, upon their transition to nursing positions, we conducted a follow‐up survey to evaluate any changes in their knowledge and application of ergonomics principles in clinical practice. The first survey was completed during their final year of studies, while the second survey included additional information regarding workplace, evaluation of ergonomic training and adherence to ergonomic principles during work in various medical institutions.

### 2.2 Participants

After receiving approval from the Institutional Board of Ethics Committee (Blind University), undergraduate student nurses towards the end of their degree were recruited through the heads of four nationally approved nursing programs and social media (e.g. student nurses' Facebook groups). Inclusion criteria were (1) lack of diagnosed chronic systemic disease or metabolic disorders, and (2) lack of lower limb swelling due to venous or lymphatic insufficiency. The sample included 133 student nurses from four nursing institutions in Israel who gave their consent to participate in the study and to be contacted 1 year later after working as a nurse practitioner in a medical institution to complete a second survey via email, such that the same sample was invited to participate in both points of time. The second survey was completed right before the COVID‐19 pandemic broke out in Israel.

This sample size, reflecting the number of participants we successfully managed to recruit across a fixed‐duration recruitment period of 2 months, indicated that with α = 0.05 and a power of β = 0.95, a sample size of *N* = 133 provides sufficient power to detect main and interaction effects (calculated in GPower 3.1; Faul et al., 2007).

Consenting participants completed a self‐administered paper questionnaire. All 133 student nurses were at the end of their fourth and final year of nursing education and had completed a course on ‘Quality Assurance and Risk Management in Nursing’. Key topics in this course encompass fundamental safety and healthcare quality concepts, the adoption of organizational culture models for safety, including patient‐centred care and teamwork, as well as the management of medical risks, research and accreditation processes to enhance patient well‐being. Another section covers the application of ergonomic principles to optimize work environments, tasks and equipment to reduce injuries, including strains and sprains, and minimize the risk of work‐related MSDs. Of the initial student nurses who completed the first survey, 45 participated in the second survey and completed a second questionnaire.

### 2.3 Research tool

The researchers developed an identical four‐section questionnaire for the two surveys, with an additional work‐related fifth section in the second survey. The questionnaire was developed based on a review of academic theory on ergonomics, work risks and occupational diseases. Age, gender, marital status, self‐rated health, exercise patterns, smoking habits, history of surgery, job title, overtime work, stress, instrumental activities of daily living (IADL) and body mass index (kg/m^2^) were included as demographic data in the study. Ergonomics awareness and knowledge were developed based on previous literature and on the Extended Nordic Musculoskeletal Questionnaire (NMQ‐E) (Dawson et al., 2009) that had been developed to investigate safety handling knowledge and practices and health which was adapted to the Israeli population and found to have moderate to high psychometric properties (Yona et al., 2020). The questionnaires were examined by five academics experts. Following equator guidelines (https://www.equator‐network.org/), several steps were taken. First a pilot study was conducted to validate the study questionnaire. Specifically, the questionnaire was distributed to five nurses studying for master's degrees for face and content validity, item relevance and comprehensibility. In response to the judges' feedback, changes were made accordingly, and the questionnaires' comprehensibility were tested in a pilot survey on a sample size of 10 student nurses and 10 nurses who were similarly characterized as the sample. Subsequently, an empirical test of the questionnaire's internal reliability was performed using SPSS (ver. 25), with Cronbach's alpha of 0.76 for the first questionnaire and 0.81 for the second. The final questionnaire is included in the supplemental materials.

The first section addressed participants' personal details, demographics and health backgrounds. Personal habits (e.g. exercise, daily driving time and hours dedicated to daily household tasks) data were collected to analyse their effects as mediating variables on pain and MSDs. The second section assessed *pain intensity* (nine body areas) using the wide‐spread visual analogue scales (VAS) from 1 (*no pain at all*) to 10 (*unbearable pain*). The data included mean values for statistical analysis. In Section 3, students indicated whether they had objective ergonomics or occupational safety *knowledge* via five questions (e.g. ‘What is the maximum cervical flexion angle when working with a patient?’), each with five multiple‐choice, concrete values options. Students' answers were recorded as correct or incorrect. The ergonomics knowledge variable was dichotomized, and the cut‐off points were set based on frequencies; a score of 0 correct answers was categorized as having poor knowledge, a score of 1–3 correct answers was categorized as having moderate knowledge and those who scored 4–5 correct answers were categorized as having good knowledge. This section also included nine questions on the students' *awareness*; subjective understanding of ergonomic issues (e.g. ‘How much do you feel you understand the term *ergonomics*?’) rated on a Likert scale of 1 (*no awareness*) to 5 (*very high awareness*). Poor ergonomics awareness was reflected by a low score. The ergonomics awareness variable was dichotomized into three groups, and the cut‐off points were set based on averages and percentages for analysis. The fourth section included 11 self‐reported musculoskeletal symptoms or disorders. The second survey administered 1 year later, consisted of identical three sections to the first questionnaire and an additional 12‐question section. Attention was given to departments in which the nurses worked and to integrating ergonomic principles in the workplace (e.g. ‘To what extent are you familiar with auxiliary or other equipment used in the workplace to transfer or move patients?’).

### 2.4 Statistical analysis

Using SPSS (ver. 26) descriptive statistics, we analysed the data to describe the participants' demographic variables and research results. Paired/Welch's *t*‐tests, Wilcoxon signed ranks, Friedman's test, one‐way analysis of variance (ANOVA) and Kruskal–Wallis tests by ranks were used to compare variables and Spearman's correlations to describe relationships between variables with a significance level of 0.05.

The study initially enrolled 133 participants, but a high rate did not complete the second survey. The reason is that a large portion of graduates decided to not work in nursing or took a year off for personal reasons; other reasons may include time constraints or lack of incentive. Welch's *t*‐test was used to compare the means between the two groups when the variances of the groups were not equal which was found in one variable—stress frequency.

## 3 RESULTS

### 3.1 Participant demographics

Most first‐survey participants (133 student nurses) were female (87%), aged 19–28 year (82%) and married (54%). Nearly 70% had no children, 14% had one child, and 5% of the female students were pregnant; almost 40% reported not driving a car, and most (62%) reported doing up to 2 h/day of housework. Half reported they exercised *not at all* or *occasionally*, and most (92%) did not smoke. The second survey involved 45 qualified nurses (34% of the first‐survey respondents completed the second survey) who had worked in the profession for about 1 year. Most (82%) were women. The percentage of married nurses increased significantly to 71%, *t*(44) = −2.07, *p* = 0.044; those without children decreased to 53% (*Z* = −2.646, *p* = 0.008); and those who reported not exercising at all or infrequently increased to 62% but was not significant. The percentage who reported not owning a vehicle barely changed (from 39.5% to 40.5%). Those with a vehicle reported driving an average of 76.5 min/day during their studies and 50 min/day during the work period, *t*(42) = 2.527, *p* = 0.015. A common (almost 21%) workplace for new nurses was the emergency room, with 16–31 weekly work hour (approximately 56%); a quarter of respondents reported more than 36 work h/week (Table 1). Twenty‐four percent of the first‐year nurse respondents reported that they attended a few work‐place sessions on safety but admitted that they never apply the principles of workplace ergonomics.

**TABLE 1:** Participant characteristics.

Variables	Student nurse (*N* = 133)		First‐year nurse (*N* = 45)		Statistic
	%	*M* ± *SD*	%	*M* ± *SD*	
*Gender*					
Male	13.0		17.8		
Female	87.0		82.2		
*Age* (*year*)					
19–28	82.0		77.8		
29–38	13.5		17.8		
39–48	4.5		4.4		
*Family status*					
Single (with parents)	27.0		13.3		
Single (live alone)	19.0		15.6		
Married	53.5		71.1		*t* = −2.072; *df* = 44; *p* = 0.044*
Widowed/divorced	0.8		0		
*Number of children*		0.64 ± 1.15		1.31 ± 1.00	*Z* = −2.646; *p* = 0.008**
0	68.9		53.3		
1	13.6		13.3		
2	7.6		22.2		
3	6.1		4.4		
4	2.3		4.4		
5	1.5		2.2		
Known pregnancy	5.3		15.6		*Z* = −2.309; *p* = 0.021*
Smoker (‘yes’)	7.6		8.9		
*Drive to work?* (*min*)		76.43 ± 93.36		49.88 ± 58.80	*t* = 2.527; *df* = 42; *p* = 0.015*
Yes	39.5		40.5		
*Daily housework time*		1.77 ± 1.06			
30 min	29.4				
1–2 h	61.5				
3–4 h	6.4				
5+ h	2.8				
BMI		23.63 ± 4.02		24.11 ± 4.23	*p* = 0.35
*Physical exercise*					*p* = 0.43
Not at all/sometimes	48.0		62.2		
Weekly	19.0		15.6		
3–2 times/week	26.8		15.6		
4 times/week	2.2		6.5		
Daily	0.7		0		
*Department*					
Internal medicine			16.3		
Emergency			20.9		
Obstetrics			2.3		
Paediatrics			7.0		
Neonatology			7.0		
Surgery			16.3		
Intensive care			11.6		
Gynaecology			4.7		
Community			9.3		
Oncology			2.3		
Operating room			2.3		
*Weekly work hour*				3.23 ± 1.26	
8–15			7.0		
16–23			27.9		
24–31			27.9		
32–36			11.6		
>36			25.6		

* *p* < 0.05.

** *p* < 0.01.

### 3.2 Health status

Hypothesis 1 that first‐year nurses would demonstrate a significant decrease in their health status (e.g. body pain and orthopaedic diagnoses) compared to when they were student nurses was supported. A significant difference was found between the two surveys in diagnostic prevalence, *t*(180) = 2.2, *p* = 0.03 (Table 2).

**TABLE 2:** Participant health status characteristics.

Pain intensity (rating)^a^	Nursing student (*N* = 133)		Nursing staff (*N* = 45)		Statistic
	%	*M* ± *SD*	%	*M* ± *SD*	
*Upper back*		2.01 ± 0.95		2.24 ± 0.98	*Z* = −2.183; *p* = 0.029*
Pain‐free	39.4		22.2		
Slight	25.8		46.7		
Average	29.5		15.6		
Strong	5.3		15.6		
*Lower back*		2.54 ± 1.05		2.91 ± 0.99	*Z* = −2.748; *p* = 0.006**
Pain‐free	21.2		8.9		
Slight	25.0		26.7		
Average	32.6		28.9		
Strong	21.2		35.6		
*Shoulder*		1.95 ± 0.97		2.11 ± 0.98	*p* = 0.17
Pain‐free	40.9		28.9		
Slight	31.8		44.4		
Average	18.9		13.3		
Strong	8.3		13.3		
*Dominant hand*		1.56 ± 0.83		2.02 ± 1.01	*Z* = −2.129; *p* = 0.033*
Pain‐free	62.6		40.0		
Slight	22.9		26.7		
Average	10.6		24.4		
Strong	3.8		8.9		
*Neck*		2.08 ± 1.03		1.91 ± 0.93	*p* = 0.69
Pain‐free	38.6		28.9		
Slight	24.2		35.6		
Average	27.3		24.4		
Strong	9.8		11.1		
*Knee*		1.85 ± 1.04		2.2 ± 0.97	*p* = 0.16
Pain‐free	51.5		26.7		
Slight	22.7		37.8		
Average	15.2		24.4		
Strong	10.6		11.1		
*Hip*		1.61 ± 086		1.87 ± 0.97	*p* = 0.13
Pain‐free	59.8		44.4		
Slight	22.7		33.3		
Average	13.6		13.3		
Strong	3.8		8.9		
*Headache*		2.20 ± 1.08		2.33 ± 1.09	*p* = 0.51
Pain‐free	34.8		28.9		
Slight	25.8		26.7		
Average	24.2		26.7		
Strong	15.2		17.8		
*No symptoms* (*0 = no*; *1 = yes*)					
Spinal column	96.2	0.04 ± 0.19	95.6	0.04 ± 0.21	*p* = 0.56
Musculoskeletal	91.7	0.08 ± 0.28	91.1	0.09 ± 0.29	*p* = 0.41
Migraine	89.4	0.11 ± 0.31	93.3	0.07 ± 0.25	*p* = 0.56
*Stress frequency*		1.88 ± 0.89		1.90 ± 0.96	*p* = 0.17
None	3.8		4.4		
Infrequently	31.8		28.9		
Sometimes	38.6		35.6		
Frequently	22.7		20.0		
All the time	2.3		4.4		
*Fatigue during work*		2.87 ± 0.86			
Very strong	7.1				
Strong	16.7				
Average	57.1				
Slight	16.7				
Very slight	2.4				

* *p* < 0.05.

** *p* < 0.01.

^a^ Likert‐scale ratings (1–10) were grouped into pain‐intensity categories: 1 (pain‐free), 2–3 (slight); 4–7 (average) and 7–10 (strong).

In the first survey, about 60% of the students reported feeling no pain in their bodies, and 5% to 32% reported pain of varying intensity. For example, students reported severe (intensity = 7–10) hand or wrist (4%), upper‐back (5%), wrist (6%), shoulder (8%), neck or knee (10%) and lower‐back (21%) pain, and 15% reported severe headaches. In the second survey, 44% of the participants reported no body pain, compared with about 60% in the first survey, *t*(44) = −2.343, *p* = 0.024.

The percentage suffering severe pain increased in some health variables: Nearly 15% of the first‐year nursing staff reported intense (7–10) upper‐back and shoulder (*Z* = −2.183; *p* = 0.029); about 9% hand and waist (*Z* = −2129, *p* = 0.033); about 36% lower‐back (*Z* = −2.748, *p* = 0.006); 7% wrist (*p* = 0.35); 11% neck (*p* = 0.69) and knee (*p* = 0.16) pain, and 18% reported severe headaches (*p* = 0.51). Spine‐related orthopaedic conditions accounted for 4.4% of the participants in the second survey, compared with 3.8% in the first (*p* = 0.56). In the second, 6.7% reported migraine compared with 10.6% in the first (*p* = 0.56), and 8.9% complained of limb disorders compared with 8.3% of the student nurses (*p* = 0.41). Moreover, stress levels were not reduced as no significant differences were found between the stress frequency student nurses experienced and that of first‐year nurses (*Z* = −1.379; *p* = 0.168). The response of ‘sometimes’ was common, for both study time (38.6%) and work time (35.6%) (Table 3).

**TABLE 3:** Differences in health status: characteristics from the two surveys.

Variable	*M* ± *SD*		*t/Z*	*df*	*p*
	Nursing students (*N* = 133)	Nursing staff (*N* = 45)			
Pain intensity	1.94 ± 0.71	2.20 ± 0.70	−2.34	44.0	0.024*
Number of diagnoses	0.08 ± 0.28	0.07 ± 0.32	2.20	180.0	0.029*
Frequency of stress	1.88 ± 0.89	1.90 ± 0.96	−1.38		0.168

* *p* < 0.05.

Hypothesis 2 was partially confirmed such that significant associations were found between pain and health‐related variables in student nurses; number of diagnoses, *r* = 0.21, *p* < 0.05, BMI, *r* = 0.24, *p* < 0.005, and driving time *r* = 0.31, *p* = 0.05, and first‐year nurses; number of diagnoses, *r* = 0.31, *p* < 0.007, and fatigue, *r* = 0.35, *p* < 0.017. (Table 4).

**TABLE 4:** Spearman correlations between pain and health‐related variables in student nurses and first‐year nurses.

*Pain*	Student nurses					First‐year nurses					
	Diagnoses (num)	Stress (freq)	BMI	Daily driving time	Physic activity	Diagnoses (num)	Stress (freq)	BMI	Daily driving time	Physic activity	Fatigue
Student nurses	0.21*	0.14	0.24*	0.31**	−0.15	0.14	−0.04	0.49**	−0.09	−0.28	−0.24
First‐year nurses	0.27	−0.16	0.18	0.36*	−0.09	0.31*	−0.19	0.25	−0.01	−0.17	0.35*

* *p* < 0.05.

** *p* < 0.01.

Additionally, a strong significant association was found between the existence of pain during study period and the existence of pain at work period (*r* = 0.61, *p* = 0.001). Furthermore, significant associations were found between diagnoses themselves during study period, between MSD's and the movement system (*r* = 0.37, *p* < 0.001) and migraines (*r* = 0.19, *p* = 0.03), between the movement system and migraines (*r* = 0.25, *p* = 0.004) and during work period pain and movement disturbances (*r* = 0.29, *p* = 0.05; Table 5).

**TABLE 5:** Spearman correlations between various health‐related variables during study and work periods.

	Study period				Work period			
	Pain	MSD's	Movement disturbances	Migraine	Pain	MSD's	Movement disturbances	Migraine
*Study period*								
Pain	–	0.09	0.26**	0.14	0.61**	0.31*	0.14	−0.09
MSD's	0.09	–	0.37**	0.19*	−0.01	−0.03	−0.05	−0.04
Movement Disturbances	0.26**	0.37**	–	0.25**	0.13	−0.05	−0.07	0.38*
Migraine	0.14	0.19*	0.25**	–	0.38*	−0.07	−0.09	0.54**
*Work period*								
Pain	0.61**	−0.01	0.13	0.33*	–	0.21	0.29*	0.15
MSD's	0.31*	−0.03	−0.05	−0.07	0.21	–	0.19	0.19
Movement Disturbances	0.14	−0.05	−0.07	−0.09	0.29*	0.19	–	0.11
Migraine	−0.09	−0.04	0.38*	0.54**	0.15	0.19	0.11	–

* *p* < 0.05.

** *p* < 0.01.

Analysed with a Spearman correlation test, the second survey results indicated a significant relationship between consecutive *standing* hours (*M* = 2.49, *SD* = 0.63) and fatigue (*r* = 0.32; *p* < 0.05) and pain intensity (*r* = 0.34; *p* < 0.01). In addition, a significant relationship was found between consecutive *walking* hours (*M* = 2.16, *SD* = 0.81) and pain intensity (*r* = 0.29; *p* < 0.05).

### 3.3 Ergonomics and health status

**FIGURE 1:**
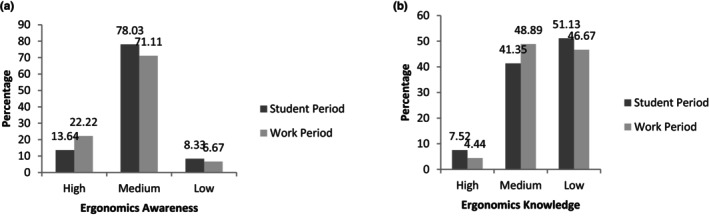
Participants' ergonomics awareness (a) and knowledge (b) in the two surveys.

Hypothesis 3 was partially confirmed. Figure 1a,b shows the distribution of responses to questions about ergonomics and safety awareness and objective knowledge of ergonomic principles in both surveys. Ergonomics knowledge level among the student nurses and first‐year nurses revealed no significant differences between the two surveys, *t*(44) = 1.16, *p* = 0.25, nor in ergonomics awareness, *t*(44) = 0.52, *p* = 0.61. Regarding ergonomic‐principle knowledge, 51% of the student nurses and 47% of the first‐year nurses chose no correct multiple‐choice answers. Most student nurses (78%) and first‐year nurses (72%) indicated medium awareness levels.

No significant differences were found in the awareness, *x* ^2^(9) = 14.89; *p* = 0.09, or knowledge levels relative to the nurses workplaces, *x* ^2^(9) = 10.68; *p* = 0.29. A one‐way ANOVA showed a significant difference between the awareness and knowledge levels among first‐year nurses, *F*(2, 42) = 4.05, *p* = 0.02, but no significant difference among student nurses (*p* = 0.07). Least significant difference analysis showed that knowledge of ergonomic principles among first‐year nurses' respondents with high awareness levels (*M* = 0.81, *SD* = 0.44) was significantly higher (*p* = 0.02) than for those with medium (*M* = −0.26, *SD* = 0.47) or low (*p* = 0.05) awareness levels. However, there was no significant difference in knowledge levels between respondents with medium compared to low awareness levels (*p* = 0.11).

**FIGURES 2:**
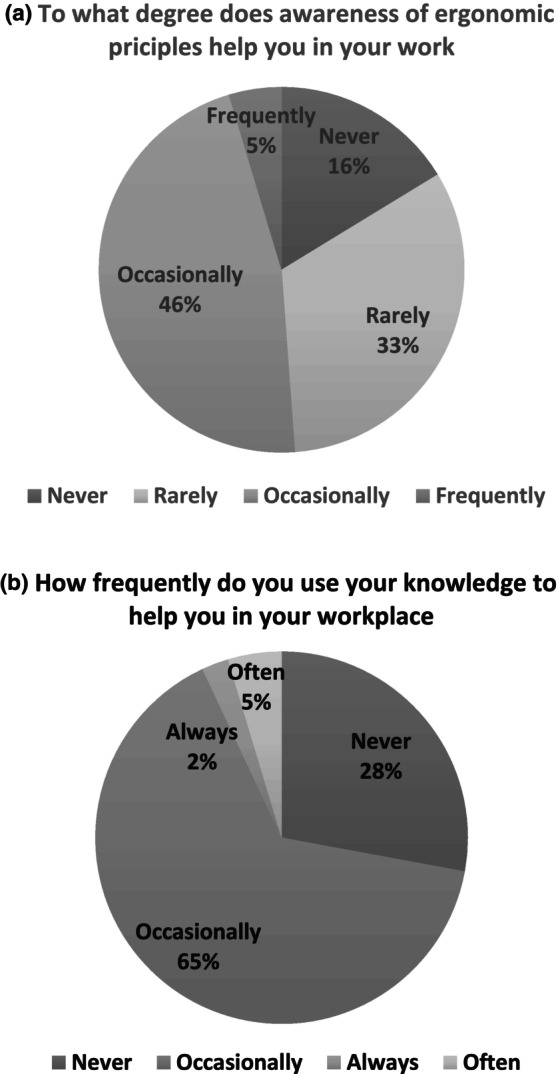
(a) Distribution (%) of answers to the degree that ergonomics awareness helps at work: 1—Largely, 2—Moderately, 3—Slightly and 4—Not at all, and (b) Frequency of use of ergonomics knowledge at work; 1 = never, 2 = occasionally, 3 = often and 4 = always.

Figure 2a,b shows the distribution of answers to questions about ergonomic principles in the workplace: 16% answered that ergonomics principles did not help at all at work, and 47% indicated they helped moderately (*M* = 2.6, *SD* = 0.82). Regarding participants' use of ergonomics knowledge, most (65%) reported *occasionally* (*M* = 2.81, *SD* = 0.63). One‐third (37%) of the respondents indicated *moderate* familiarity with adapted and patient‐transfer equipment (*M* = 3.07, *SD* = 1.28), 33% rated *moderate* attention to body mechanics during work, (*M* = 3.12, *SD* = 1.18), and only 30% reported *very good* familiarity with the location of assistive devices in their workplaces (*M* = 2.23, *SD* = 0.97). Nearly half (47%) of the respondents walked continuously for 4 h during a working day; one‐third (37%) walked (*M* = 2.16, *SD* = 0.81), and more than half (56%) stood (*M* = 2.49, *SD* = 0.63) for more than 5 consecutive working hour.

Analysis of the second survey indicated a significant main effect in pain intensity relative to ergonomics *awareness* and *knowledge*, thus supporting Hypothesis 3. There was a significant difference between the pain‐intensity averages (high: *M* = −0.56, *SD* = 0.44; average: *M* = 0.02, *SD* = 0.54; low: *M* = 0.92, *SD* = 0.94), with increased pain intensity and decreased awareness, *F*(2,42) = 13.1, *p* < 0.001, η^2^ = 0.61, and knowledge, *F*(4,42) = 4.98, *p* = 0.003, η^2^ = 0.12 (Table 6).

**TABLE 6:** Interaction between the ergonomics awareness and the pain intensity.

Pain intensity	Level of awareness of the field of ergonomics						
	*M* ± *SD*						
	High	Average	Low	d*f*	*F*	η^2^	*p*
First‐year nurse	−0.56 ± 0.44	0.02 ± 0.54	0.92 ± 0.94	2,42	9.21	0.61	0.000**
Student nurse	2.03 ± 0.80	1.96 ± 0.71	1.56 ± 0.41	2,129	1.77	0.03	0.170

** *p* < 0.01.

The analysis also indicated an interaction effect between ergonomics awareness and knowledge, such that there was a significant difference between the average pain intensity among the nursing staff relative to their awareness and knowledge levels, *F*(4,42) = 2.98, *p* = 0.033, η^2^ = 0.41. No significant difference was found between the same variables during the *nursing student* period (*p* = 0.17; Table 7).

**TABLE 7:** Effects of awareness and knowledge variables on pain during nursing work.

Variable	df	*F*	η^2^	*p*
(A) Ergonomics awareness level	2	13.1	0.61	0.000**
(B) Ergonomics knowledge level	4	4.98	0.12	0.003**
A × B	4	2.98	0.414	0.033*

* *p* < 0.05.

** *p* < 0.01.

An ANOVA analysis showed that there is a significant difference in the level of ergonomics knowledge between those with higher ergonomics awareness among the first‐year nurses [*F*(2,42) = 4.05, *p* = 0.02], whereas there was no significant difference between the same respondents who answered during their nursing studies (*p* = 0.07). From the LSD analysis, it appears that knowledge of ergonomic principles is significantly higher among first‐year nurses with high awareness of the patient (*M* = 81, *SD* = 0.44) compared to those with moderate awareness (*SD* = 0.47, *M* = 0.26) but not significantly different between those with moderate or low awareness. Intriguingly, in first‐year nurses, no significant differences were found between those with higher or lower ergonomics knowledge/awareness in their number of medical diagnoses, the existence of migraine, fatigue or MSDs. This remains to be further investigated in larger groups of participants and statistically analysed using demographic and health mediators.

Hypothesis 4 *was confirmed*. Nearly 40% of nursing students indicated that the course ‘Quality Assurance and Risk Management in Nursing’ did not prepare their ability to identify risk factors in the context of bodily pain or prepared them to a low extent (28%) (*M* = 3.81, *SD* = 1.22). Sixty percent of the first‐year nurses answered the same question by stating that the course had a significant or moderate impact (*M* = 3.24, *SD* = 1.38); however, Friedman's test showed that their ergonomics preparedness was significantly lower [*x* ^2^ (2,45) = 10.177; *p* = 0.006]. In addition, when asked about the level of training in ergonomics in the medical institution where they work, their responses normally distributed, revealed that 16% did not receive any training, 19% received minimal training, 33% received moderate training, and nearly 26% received adequate training. In other words, half of the respondents indicated that the impact of training was moderate or less.

Findings showed significant correlations between the satisfaction of the efficiency of ergonomic training in the nursing program as perceived by first‐year nurses and adherence to ergonomic principles during nursing work, *r* = 0.40; *p* = 0.005, attention to body mechanics (*r* = 0.32; *p* = 0.028) and ergonomics awareness (*r* = 0.33; *p* < 0.05).

## 4 DISCUSSION

The main purpose of this study was to compare the health status of student nurses to that of first‐year nurses and examine if ergonomics knowledge and awareness through educational preparedness affects their health status. Firstly, final‐year student nurses' awareness of ergonomic principles and knowledge of medical ergonomics was evaluated. Following graduation, after they worked for 12 months, their awareness and knowledge levels were reexamined and compared to results from their student‐nursing period. An additional comparison examined their health status changes over the same period.

The second (follow‐up) survey showed that more than two‐thirds (78% of students and 72% of staff) indicated a medium level of ergonomics awareness. Their knowledge of ergonomic principles was lower than expected (51% vs. 47%). About 60% of the nursing staff did not know the ergonomics concept at all, similar to results from other countries (Braeckman et al., 2017; Dąbek et al., 2019; Kneafsey et al., 2012; Saremi et al., 2019) where insufficient ergonomics awareness levels exist.

Most (63%) answers to multiple‐choice questions on ergonomic‐principle knowledge (e.g. maximum body angle in front of patients, neck flexion during work, lift weight, and consecutive standing and walking hours during work) indicated an insufficient understanding of the importance of ergonomics at the worksite. The percentage of compliance with the principles ranged from 46.5% responding *moderate* to 32.5% responding *less than moderate*. Most (65%) respondents reported expressing their knowledge of ergonomics *sometimes*. Less than 25% stated that they paid attention to body mechanics, and only one‐third were familiar with assistive devices and equipment for transferring patients. Finally, more than half of the nursing staff stood for over 4 h and walked for over 5 h consecutively throughout the workday.

Understanding ergonomic principles is crucial. Physical risk factors in ergonomics, such as using heavy manual equipment and repetitive or strenuous posture and movements, lead to muscle fatigue. Fatigue, in turn, leads to back, neck and upper‐extremity pain, resulting in orthopaedic injuries over time. In addition, neck–muscle pain and spasms can lead to aches in various parts of the head and face (Pheasant, 2003).

This study's results indicate an increasing trend in musculoskeletal pains and injuries with increased work exposure. These results are somewhat paradoxical: One would anticipate work‐related experiences enhancing wellness. Moreover, the concurrence between pain intensity and awareness of ergonomic principles may indicate harmful working conditions (noncompliant with ergonomic principles). Possible explanations for this adverse working environment may be inadequate administrative control, lack of personnel control or insufficient training. Nevertheless, the findings reinforce ergonomics awareness implications for ergonomic knowledge among nursing staff.

The relationship between ergonomics awareness and compliance with ergonomic principles in the work environment supports the need for more effective work behaviours regarding risk prevention and occupational health. Noncompliance with the principles leads to musculoskeletal and migraine disorders, reflected in the relationship between hours of prolonged standing or walking at work and pain‐intensity and fatigue levels.

This study's results support the work of other researchers who claimed that noncompliance with ergonomic principles, such as prolonged standing, poor bending movements and extended working hours, cause MSDs and body aches among nursing staff (Dąbek et al., 2019; Kurowski et al., 2017). Other studies found that standing or walking for more than 3 h during a workday and standing for over 80% of a shift can cause lower‐back and leg pain (Sezgin & Esin, 2018). They noted that monotonous work, heavy lifting (Karahan et al., 2009), patient‐care tasks in general and inappropriate body postures during demanding activities (Reed et al., 2014) caused long‐term MSDs (Backåberg, 2016).

Additional results of this study established several significant relationships between variables related to the respondents' health. A direct relationship was found between pain intensity and musculoskeletal or migraine disorders diagnoses; a significant relationship was found between fatigue levels during work and the existence of pain. Furthermore, the results establish a relationship between pain during nursing studies and pain intensity during nursing work and significant positive relationships between several diagnoses. Unfortunately, one can surmise that the health condition of new nursing staff can be expected to worsen due to their working circumstances.

The demands of the modern medicine world, including increasing workloads and hospital personnel shortages, result in physical and cognitive pressure that can lead nursing staff to experience occupational fatigue and burnout and leave the profession (Ji et al., 2019). Shift work, specifically night shifts (Fratissier et al., 2021), has been associated with sleep problems and fatigue, and nurses' cognitive, emotional and physical manifestations of fatigue often have additional health consequences (Axelsson & Kecklund, 2016; Brzozowski et al., 2021; Okuhara et al., 2021). A scoping review (Gifkins et al., 2020) emphasizes the intricate challenges associated with shiftwork scheduling for nurses, as it disrupts sleep patterns, circadian rhythms and ultimately results in reduced alertness, inattentiveness, workplace conflicts and diminished performance, with long‐term fatigue negatively affecting both nurses' well‐being and their own safety as well as their patient's safety.

Lipscomb et al. suggested that preventing MSDs requires a system‐level approach to work‐shift scheduling to reduce exposure to demanding work conditions and promote healthy work–rest patterns (Lipscomb et al., 2002, 2004).

Given that fatigue at work likely increases pain and MSDs, it is unfortunate that only 12% of nursing staff respondents in this study regularly exercise during their leisure time. The US Department of Health and Welfare considered exercise a priority to advance wellness within the population significantly. Similarly, OECD countries adopted physical‐activity guidelines, stating that regular physical activity could produce long‐term health benefits (Physical Activity Guidelines Advisory Committee, 2018). Previous studies revealed a statistically significant association between BMI values and pain and health‐related variables in nurses, but not with knowledge of ergonomic principles or adherence to work ergonomics. However, higher BMI values have been attributed to the observed impact of noncompliance with ergonomic principles on musculoskeletal pain (Kołcz et al., 2020; Mikiciuk et al., 2012; Tantawy et al., 2017). Furthermore, lower regular aerobic and muscle‐strengthening physical activities were associated with higher BMI and interestingly with lower musculoskeletal pain (Nam et al., 2018). This finding suggests that having musculoskeletal symptoms negatively affects nurses' physical activity, but experiencing more or greater symptoms may affect their motivation to perform physical activity to manage their symptoms or prevent further injuries.

It is encouraging to note that global nursing education curricula include ‘quality assurance and risk management in nursing’. However, there is still an insufficient emphasis on physical exercise as part of ergonomic training among nursing staff. In this study, the students' and nursing staff's questionnaire responses indicated they felt that not all nursing and medical institutions have proper ergonomics courses. Ketelaar et al. (2015) concluded that new nurses in the Netherlands needed more knowledge and support to improve their health and functioning at work. Student nurses did not receive sufficient knowledge of occupational health during their nursing studies and had insufficient awareness of the risks associated with ergonomic dysfunction. In practice, new nurses are attentive to patients' needs and make every effort in patient care. Regrettably, new nurses do not attend to their own needs for proper body mechanics when lifting or operating medical equipment.

Furthermore, nurses often are unaware of the availability or location of various lifting aids and equipment. Staff training and coaching on patient transfers and manual mobility are essential components of an ergonomic‐safety improvement program for nurses and patients. However, training nurses to use biomechanical principles alone is insufficient to reduce their physical load during patient transfers or the risk of injury (Driessen et al., 2011; Nelson, 2006). Durham's (2012) 30‐year longitudinal study demonstrated that manual patient care and body mechanics were insufficient to maintain medical staff wellness. They found care using assistive devices during patient transfers to be a crucial variable in reducing MSDs in nursing staff.

Nevertheless, nursing education institutions continue to teach outdated treatment methods based on ‘correct’ body mechanics, such as basic two‐person methods for lifting and transferring patients. Alone, such methods could put staff health at risk. A national survey found that most curricula included outdated manual techniques, taught reliance on body mechanics to reduce the risk of musculoskeletal injuries and made use of nonergonomic aids such as draw sheets (Powell‐Cope et al., 2018).

An ergonomic risk‐management program also should teach ergonomic principles, cognitive–behavioural and workplace‐related interventions, and social support and emphasize using adaptive equipment (Black et al., 2011; Durham, 2012).

The work organization is responsible and able to prevent MSDs through ergonomics exposure and by alleviating biomechanical loads, which also can lessen stress (Lipscomb et al., 2004; Trinkoff et al., 2006). Multifaceted ergonomic interventions need to incorporate individual level training and administrative intervention (e.g. 10 min daily stretching exercises break) with engineering interventions (i.e. lifting and usage of auxiliary devices) to be effective (Coskun Beyan et al., 2020). The US Occupational Safety and Health Administration's ergonomic guidelines suggest that employee health can be maintained by planning spacious and comfortable work environments, ensuring movement and exercise throughout the day, and erecting displays and control signs. They promote working in a neutral position, reducing the use of excessive force, planning the workspace to ensure work equipment is within reach, adjusting work positions to workers' heights, avoiding unnecessary movements, reducing static loads and minimizing fatigue (US Department of Labor, 2021).

The Panel on Musculoskeletal Disorders and the Workplace (National Research Council, 2001) suggested a conceptual model which depicts the potential factors contributing to musculoskeletal disorders, encompassing influences from both the environment (i.e. workplace) and the person (i.e. individual characteristics within the person). The model identifies factors which encompass external physical loads and organizational factors, while individual factors are related to the individual's biology, psychology and social context which include age, gender, health habits, comorbidities, genetic predispositions and non‐workplace physical activities like exercise and household work. The model is compatible with the International Classification of Functioning, Disability and Health (ICF), a WHO‐developed health model used to classify health and health‐related conditions, taking into account the impact of health on an individual's functioning and well‐being. It comprises two main components: (1) functioning and disability, focusing on body functions, activities and participation; and (2) contextual factors, including environmental and personal factors. The concept of disability, as defined by the WHO's International Classification of Functioning (ICF), encompasses not just physical impairments but also limitations in activities and participation, bridging medical and social models of disability. Workers with musculoskeletal disorders often experience participation limitations (Krishnan et al., 2021) poor return‐to‐work rates and significant socioeconomic costs, influenced by psychosocial factors. The social consequences of occupational injuries and illnesses can lead to reduced income, unemployment, stress, depression and other negative impacts, with a more significant burden on women in healthcare professions (Heerkens et al., 2017). Prioritizing the creation of an ICF ontology that accommodates work‐related environmental factors and personal factors is essential for identifying ergonomic principles which utilize person‐environment‐occupational concepts to equip nurses in the field, educators, allied healthcare professionals and employers' tools for promoting improved ergonomics educational preparedness and interventions which can impact healthy work participation of nurses at their workplace.

### 4.1 Limitations

This study's limitations lie in that the target participants who participated in the second session included 45 participants from the initial 133 which may bias the study results. Even though most non‐completers were graduates who did not immediately join the work force—which may have impacted reluctance to initially participate in the first survey—the variables comparing characteristics of completers and non‐completers are unknown. Nevertheless, the authors were able to analyse data from the remaining participants. Sample recruitment was both through the schools and the social media, in anticipation of obtaining a larger sample for the initial survey. The relatively small sample may potentially be attributed to the students' activities across various platforms that may differ from those employed by researchers. There also may be consequences of organizational and cultural work aspects that differ between countries which may limit the generalizability of the results. Additionally, although the study included a relatively wide range of medical departments, it did not encompass the complete range; thus results should be interpreted with caution.

## 5 CONCLUSIONS

The science of ergonomics can support a suitable work environment for employees by increasing physical adaptations. Because ergonomics improves user comfort, maintains health and increases teamwork productivity (Pheasant, 2003), failure to adopt ergonomic principles lowers productivity and increases work discomfort. There is a close relationship between ergonomic‐principle compliance in work settings and occupational health among nursing staff. One reason for noncompliance with ergonomic principles is the lack of awareness and knowledge of them (Karahan et al., 2009; Sezgin & Esin, 2018). Student nurses in this study displayed insufficient awareness and weak knowledge of ergonomic principles. Following a year of experience working in nursing, their awareness and knowledge levels hardly changed, but their states of health declined.

Two major conclusions emerge from these results: To prevent long‐term health damage, every nursing staff member who undergoes professional training should (1) be skilled at working according to the ergonomic principles and (2) take responsibility for their health by adhering to those principles. State policy should legally require broadly and deeply incorporating medical ergonomics into basic nursing curricula. Given the increased life expectancy and promotion of population health, the number of nursing staff in hospitals is higher than ever. It is paramount to preserve their and other healthcare professionals' wellness and quality of life by establishing a culture of promoting ergonomics that can benefit the entire health system. Research in nursing education programs suggests partnering with faculty in physical and occupational therapy departments, clinical partnering and working with equipment vendors—to better incorporate ergonomic principles and practices into nursing curricula (Powell‐Cope et al., 2018; Richardson et al., 2018; Salman et al., 2022). Educational preparedness plays a significant role in facilitating ergonomics awareness in nurses. By providing education and training on ergonomics, nurses can gain an understanding of how to prevent injuries and strain on their bodies while performing their job duties. This, in turn, can help reduce work‐related injuries and improve the overall health and well‐being of nurses. This includes learning how to adjust equipment and workstations, as well as how to use proper body mechanics to prevent injuries. They can identify potential hazards and take steps to minimize or eliminate them, such as improving lighting, adjusting the height of equipment and using appropriate lifting techniques. Further high‐quality research is required to ascertain the development, evaluation and sustainability of educational ergonomics training and to determine the long‐term benefits to the health of nursing staff (Salman et al., 2022).

### RELEVANCE TO CLINICAL PRACTICE

The contribution of this study to the wider global clinical community is to illuminate the association between the lack of awareness and knowledge of ergonomic principles existing in the nursing staff and its association to long‐ term health damage. Notwithstanding, strong emphasis should be placed on teaching ergonomic principles during nursing education and reinforced during the first‐year training process. Ergonomic training in nursing education can also help nurses implement ergonomic strategies in their daily work routine. This can include using proper posture, taking frequent breaks and stretching exercises to reduce pain, musculoskeletal injury and fatigue. Medical institutions should ensure that nursing staff are skilled at working according to ergonomic principles to prevent long‐term health disorders and reduce drop‐out.

## AUTHOR CONTRIBUTIONS

MH has contributed to the research methods, structure of the paper and editing. EL has contributed to the concept, data collection, and both have equally contributed to the data analysis and writing.

## FUNDING INFORMATION

This study has not received funding.

## CONFLICT OF INTEREST STATEMENT

The authors have no conflict of interest.

## ETHICS STATEMENT

The research received ethical approval from the IRB (REDACTED).

## INFORMED CONSENT

All participants signed an informed consent document.

## Supporting information


Data S1.

## Data Availability

The data that support the findings of this study are available on request from the corresponding author. The data are not publicly available due to privacy or ethical restrictions.
