# Supplementary material for: Health status and ergonomics education: A comparison between student nurses and first‐year nursing staff
Source: Nurs Open. 2024 Jul 11;11(7):e2239. doi: 10.1002/nop2.2239 (PMC11237340; doi:10.1002/nop2.2239)
Supplement: Supplementary file 1 — Data S1. [file NOP2-11-e2239-s001.docx]

Supplementary materials

**Health Status and Ergonomics Education: A Comparison between Student Nurses and First-Year Nursing Staff**

Time 1. Dear nursing student (first survey),

Thank you for participating in the study. This questionnaire is intended for nursing students towards the end of their 4^th^ and final year of nursing education. The questionnaire includes several sections which relate to your health and everyday occupations as well as additional future work-related questions. The average time to complete it is about 20 minutes. As you have provided your consent, we will contact you a second time after a work period of 12 months in healthcare institutions to complete a second survey.

Time 2. Dear Nurse (second survey),

Thank you for participating in the study. This second questionnaire is part of the research you have given your consent to participate in and fill out. The questionnaire is intended for nursing staff who have been working for 12 months. The questionnaire includes several sections which relate to your health and everyday occupations as well as additional work-related questions. The average time to complete it is about 25 minutes.

**Section 1 (Students and nurses)**

The following questions are related to personal and demographic information:

1. Gender:

1) Male 2) Female 3) Prefer not to answer

1. Age:

1) 19-28 2) 29-38 3) 39-48 4) 49 or older

1. Marital status:

1) Single, living alone 2) Single, living with parents 3) Married/Living with partner 4) Divorced/ widowed

1. Number of children:

1) 0 2) 1 3) 2 4) 3 5) 4 6) 5 or more

1. Known pregnancy:

1) No 2) Yes 3) Unknown

1. Do you smoke?

1) Yes 2) No

1. How many days a week do you engage in physical activity for more than 30 minutes?

1) Never 2) Occasionally 3) Once a week 4) 2-3 times a week 5) 4 or more times a week

1. How often do you feel psychological stress?

1) All the time 2) Often 3) Sometimes 4) Rarely 5) Never

1. On average, how many hours a day do you spend on household chores such as laundry, cleaning,

dishwashing, etc.?

1) Less than half an hour 2) 1-2 hours 3) 3-4 hours 4) more than 5 hours

1. BMI

Height:

Weight:

1. Driving

Driving hours (if any) per day for commuting to work:

Driving hours (if any) per day excluding commuting to work:

**Section 2 (Students and nurses)**

The second section of the questionnaire deals with acute or chronic pain (if present at all). Please circle the answer that best characterizes the current intensity of your pain on a scale of 1 to 10, where 1 represents no pain at all and 10 represents unbearable pain.

No Pain Unbearable Pain

1. Up to what intensity do you experience upper back pain? 1 2 3 4 5 6 7 8 9 10
2. Up to what intensity do you experience lower back pain? 1 2 3 4 5 6 7 8 9 10
3. Up to what intensity do you experience shoulder pain? 1 2 3 4 5 6 7 8 9 10
4. Up to what intensity do you experience hand pain? 1 2 3 4 5 6 7 8 9 10
5. Up to what intensity do you experience pain in the base of your thumb? 1 2 3 4 5 6 7 8 9 10
6. Up to what intensity do you experience neck pain? 1 2 3 4 5 6 7 8 9 10
7. Up to what intensity do you experience knee pain? 1 2 3 4 5 6 7 8 9 10
8. Up to what intensity do you experience hip pain? 1 2 3 4 5 6 7 8 9 10
9. Up to what intensity do you experience headache? 1 2 3 4 5 6 7 8 9 10

**Section 3 (Students and nurses)**

The third section of the questionnaire refers to concepts related to ergonomics and safety:

1. How well do you feel you understand the concept of "ergonomics"? 1) Very well 2) Well 3) Approximately 4) Not very much 5) Not at all
2. What is your awareness level regarding physical risks among a nursing team? 1) Very aware 2) Aware 3) Approximately 4) Not very much 5) Not at all
3. What is your awareness level of "straining posture"? a) 1) Very aware 2) Aware 3) Approximately 4) Not very much 5) Not at all
4. What is your awareness level of "heavy manual lifting"? 1) Very aware 2) Aware 3) Approximately 4) Not very much 5) Not at all
5. What is your awareness level of "repetitive motion"? 1) Very aware 2) Aware 3) Approximately 4) Not very much 5) Not at all
6. What is your awareness level of "frequent or sustained object handling"? 1) Very aware 2) Aware 3) Approximately 4) Not very much 5) Not at all
7. How well do you understand the risk factors involved in moving, lifting, and transferring patients? 1) Very well 2) Well 3) Approximately 4) Not very much 5) Not at all
8. What is your awareness level of using a manual lift during patient transfer? 1) Very aware 2) Aware 3) Approximately 4) Not very much 5) Not at all
9. To what extent has course content improved your ability to identify risk factors in nursing work in the context of your body pain? 1) To a great extent 2) To a moderate extent 3) To some extent 4) To a small extent 5) Not at all
10. To your knowledge, what is the recommended maximum body angle when standing in front of the patient during treatment? 1) 15-25 degrees 2) 30-35 degrees 3) 40-45 degrees 4) 50-65 degrees 5) 70-90 degrees
11. To your knowledge, what is the recommended maximum cervical bending angle when lifting patients? 1) 0-10 degrees 2) 15-25 degrees 3) 30-35 degrees 4) 40-45 degrees 5) 50-65 degrees
12. To your knowledge, what is the recommended maximum bending angle of the hands during treatment? 1) 15-25 degrees 2) 30-35 degrees 3) 40-45 degrees 4) 50-65 degrees 5) 70-90 degrees
13. To your knowledge, what is the recommended maximum weight that can be lifted during work? 1) Less than 1 kg 2) 1-3 kg 3) 3-5 kg 4) 5-8 kg 5) 8-12 kg 6) 12-20 kg 7) More than 20 kg
14. To your knowledge, how many consecutive hours of walking do you think can be done during a work shift? 1) Less than 2 2) 2-4 3) 4-6 4) 6-8 5) more than 8

**Section 4 (Students and nurses)**

The fourth section seeks to determine whether you suffer from chronic diseases or problems related to the musculoskeletal system, back, and head (if any exist). Please circle the most appropriate answer between "Yes"(diagnosed), "No", "Other" (if you suffer), or "Undiagnosed” (in the midst of testing):

1. Do you have a herniated or protruding disc in your spine? 1) Yes 2) No 3) Other 4) Undiagnosed
2. Do you have spondylolisthesis? 1) Yes 2) No 3) Other 4) Undiagnosed
3. Do you have sciatica? 1) Yes 2) No 3) Other 4) Undiagnosed
4. Do you have visceral diseases? 1) Yes 2) No 3) Other 4) Undiagnosed
5. Do you have carpal tunnel syndrome? 1) Yes 2) No 3) Other 4) Undiagnosed
6. Do you have circulatory or vein problems? 1) Yes 2) No 3) Other 4) Undiagnosed
7. Do you have inflammation of the tendons of the legs? 1) Yes 2) No 3) Other 4) Undiagnosed
8. Do you have joint inflammation? 1) Yes 2) No 3) Other 4) Undiagnosed
9. Do you have migraines? 1) Yes 2) No 3) Other 4) Undiagnosed
10. Do you have other movement system-related problems?

1) Yes 2) No 3) Other 4) Undiagnosed

1. Do you have other neck-related problems? 1) Yes 2) No 3) Other 4) Undiagnosed

**Section 5 (for nurses only)**

This section addresses your workplace:

1. In which department do you work? 1) Internal medicine 2) Emergency 3) Geriatrics 4) Paediatrics 5) Cardiology 6) Surgery 7) Intensive care unit 8) Rehabilitation 9) Other
2. How many hours per week do you work on average? 1) Less than 8 2) 8-15 3) 16-23 4) 24-31 5) 32-36 6) More than 36
3. To what extent are you familiar with the assistive devices or other equipment used to transfer or move the patient in your workplace? 1) Not at all familiar 2) Slightly familiar 3) Somewhat familiar 4) Moderately familiar 5) Extremely familiar
4. How many consecutive hours do you work on average during a workday? 1) Less than half an hour 2) 1-2 3) 3-4 4) More than 5 hours
5. How many consecutive hours do you walk during a workday on average? 1) Less than half an hour 2) 1-2 3) 3-4 4) More than 5 hours
6. To what degree does awareness of ergonomics help you in your work? 1) Largely helps 2) Helps very much 3) moderately helps 4) Slightly helps 5) Does not help
7. How often do you use ergonomic knowledge during your work? 1) Always 2) Often 3) Occasionally 4) Rarely 5) Never
8. What is your level of fatigue during your work? 1) Very high 2) High 3) Moderate 4) Light 5) Very light
9. What, in your opinion, is your level of ergonomic training you received before starting your work at the medical institution? 1) Excellent 2) Very good 3) Good 4) Fair 5) Poor
10. What is your level of attention to your body mechanics during lifting or moving patients or providing care to them? 1) Excellent 2) Very good 3) Good 4) Fair 5) Poor
11. How familiar are you with the location and availability of assistive devices and equipment in your institution? 1) Not at all familiar 2) Slightly familiar 3) Somewhat familiar 4) Moderately familiar 5) Extremely familiar
